# Utility of repeat testing for COVID-19: Laboratory stewardship when the stakes are high

**DOI:** 10.1017/ice.2020.397

**Published:** 2020-08-03

**Authors:** Lindsey M. Rearigh, Angela L. Hewlett, Paul D. Fey, M. Jana Broadhurst, David M. Brett-Major, Mark E. Rupp, Trevor C. Van Schooneveld

**Affiliations:** 1 Division of Infectious Diseases, University of Nebraska Medical Center, Omaha, Nebraska; 2 Division of Pathology and Microbiology, University of Nebraska Medical Center, Omaha, Nebraska; 3 Department of Epidemiology, University of Nebraska Medical Center, Omaha, Nebraska

## Abstract

As the coronavirus disease 2019 (COVID-19) continues to circulate, testing strategies are of the utmost importance. Given national shortages of testing supplies, personal protective equipment, and other hospital resources, diagnostic stewardship is necessary to aid in resource management. We report the low utility of serial testing in a low-prevalence setting.

Rapid and accurate testing for severe acute respiratory coronavirus virus 2 (SARS-CoV-2) during the coronavirus disease 2019 (COVID-19) pandemic is essential to limit transmission and to aid in appropriate management of hospitalized patients. Understanding of the performance of COVID-19 nucleic acid testing in clinical settings is evolving. Many false-negative reverse transcription polymerase chain reaction (rt-PCR) tests have been reported, raising concerns regarding infection control and patient placement.^1–3^ Apprehension regarding transmission prevention along with lack of data on test performance have led to the ordering of serial tests for persons under investigation. We assessed the value of repeat testing for COVID-19 after an initial negative result to evaluate test performance and to determine whether repeat testing is necessary.

## Methods

We retrospectively reviewed inpatient and outpatient test results among patients who presented to our academic medical center in Omaha, Nebraska, from March 10 through April 28, 2020. Patients with a negative or inconclusive COVID-19 rt-PCR test followed by at least 1 additional test were included. Patients who had testing separated by >14 days were excluded because there was significant risk of new exposure during that period, prompting testing. Basic demographic information and clinician indication for testing were collected via electronic medical record review. Testing was performed with the cobas SARS CoV-2 on the Roche 6800 platform, and a second laboratory developed an RT-PCR test (NE_CoV_19). The cobas SARS CoV-2 uses primers and probes that detect the ORF1a and E genes whereas the NE_CoV_19 assay detects the E and N genes. Using the NE_CoV_19 assay, if the E-gene was detected, the same RNA sample was retested with the E-gene and N-gene as targets. If either the E-gene or the N-gene or both were detected, the test was positive. However, if gene targets were inconsistently identified and neither the E-gene nor the N-gene was not detected by the repeat test, the test was inconclusive.

## Results

In total, 275 patients with initially negative or inconclusive test results (94% negative, 6% inconclusive) underwent at least 1 additional test. Results of the second test were 98.5% (271 of 275) negative, 0.5% positive (1 of 275), and 1% inconclusive (3 of 275). Furthermore, 40 patients (14.5%) underwent a third test, with 97% negative results (39 of 40) and 3% positive results (1 of 39). Also, 10 patients (1.5%) underwent >3 tests, with no positive results. Patient characteristics, indications for testing, and sample sources are outlined in Table 1. Median time from symptom onset to initial test was 3 days, with 69% of initial tests performed in the first week of illness and 17% performed on asymptomatic patients (primarily preprocedural screening). All samples were obtained from the respiratory tract. The most testing was performed via nasopharyngeal (NP) swab, although lower respiratory tract (LRT) sampling was more common with repeat testing. Median time between testing was longer among second and third tests compared to first and second tests (3.8 days vs 1 day). The most common indication for repeat testing was signs and symptoms concerning for COVID-19 infection or evidence of LRT infection on imaging via either chest CT or x-ray: 58% for the second testing indication and 50% for the third testing indication.

**Table 1.** Population Characteristics and Testing Indication

Characteristic	No. (%) (N = 275)
Median age, y (range)	62 (1–94)
Male sex	143 (58)
Median time between 1^st^ and 2^nd^ tests, d (range)	1.0 (0–14)
Median time between 2^nd^ and 3^rd^ tests, d (range)	3.8 (1–14)
**Time to initial testing from symptom onset**	
1–7 d	190 (69)
8–14 d	27 (10)
>14 d	10 (4)
Screening d^a^	48 (17)
**2^nd^ test indication^b^**	
Symptoms suggestive of COVID-19 infection or evidence of LRTI on imaging	158 (58)
Immunosuppressed patient^c^	55 (20)
High-risk exposure^d^	27 (10)
Additional screening needed^a^	69 (25)
Other^e^	21 (8)
**3^rd^ test indication^b^ (N = 40)**	
Symptoms suggestive of COVID-19 or evidence of LRTI on imaging	20 (50)
Immunosuppressed patient^c^	3 (7)
High-risk exposure^d^	2 (5)
Additional screening needed^a^	19 (48)
**Sample source**	
1^st^ test (N = 275)	
Nasopharyngeal	272 (99)
Tracheal aspirate/sputum	3 (1)
2^nd^ test (N = 275)	
Nasopharyngeal	255 (93)
Tracheal aspirate/sputum	20 (7)
3^rd^ test (N = 40)	
Nasopharyngeal	31 (77)
Tracheal aspirate/sputum	9 (23)

a Screening was required for planned procedures, for patients residing in a group setting, and for those with frequent contact with healthcare facilities. Additional screening was required prior to hospital discharge.

b Patients could have >1 indication for 2^nd^ and 3^rd^ testing.

c Included patients on immunosuppressive medications, transplant recipients, patients with active cancer on chemotherapy, and patients with HIV with CD4 count <200.

d Includes exposure to a person who is a known positive or currently under investigation for COVID-19.

e Reason not documented or clinician requesting additional testing.

## Discussion

We found that repeat testing in patients who had initially tested negative had very low utility. Of 275 patients tested, 2 tested positive from an NP swab on serial testing after an initial negative result. However, there was a high clinical suspicion for COVID-19 infection in both cases due to bilateral ground glass consolidations seen on chest CT. Patient 1 presented 6 days from symptom onset after traveling in a high-prevalence area. Patient 2 presented 5 days from symptom onset after a high-risk exposure to a known COVID-19–positive patient. Decreased sensitivity in NP sampling has been demonstrated in studies, particularly later in the disease course and if pneumonia is present.^4,5^ This decreased sensitivity is thought to be due to waning viral load levels in the upper respiratory region.^6–8^

Diagnostic stewardship is essential to improving the efficiency of the testing process and to conserve testing supplies and personal protective equipment (PPE) given critical national shortages.^9^ Patient flow and bed availability are also affected by duplicate testing, which can delay patient transfer out of isolation rooms as well as increase PPE utilization while test results are pending. Further patient work-up and definitive diagnoses can also be delayed due to physician anchoring and confirmation biases. Equipment such as swabs and reagents to perform testing are limited, emphasizing the need to safely limit testing particularly in low-prevalence areas to allocate resources were more testing is necessary.

Based on our findings, we modified our testing recommendations to state that a single appropriately obtained NP swab performed within the first 7 days of illness is generally adequate to rule out COVID-19. If clinical suspicion persists due to high-risk exposure or classic clinical presentation, additional testing should be obtained. Patients presenting with pneumonia may benefit from repeat testing utilizing specimens from the LRT. Preference for collection of LRT specimens in patients with pneumonia is in line with both the World Health Organization and Centers for Disease Control recommendations.^10,11^ Our guideline revision was implemented via education of our COVID teams and ID physicians. Our current practice of determining the need for additional testing involves patient review by a multidisciplinary team, including an infectious diseases physician with experience in managing patients with COVID-19. The ID physician leading this team generally determines whether additional testing is needed after input from the primary team.

Limitations of this study include a relatively low COVID-19 community prevalence (average 7% of tests positive), which limits its generalizability to high-prevalence settings. Testing in our facility utilized 2 assays that demonstrated similar performance for COVID-19 detection, but these results may not be applicable to other assays. Repeat testing was not systematically employed and cases may have potentially been missed, but at this time we are unaware of any missed COVID-19 cases in our facility.

In conclusion, we found little benefit of repeat testing in patients who presented early in their illness. We have adopted diagnostic testing stewardship, limiting testing to a single nasopharyngeal swab sample in most cases. Facilities should continually evaluate test performance and utilization to maximize the value of such tests, especially when faced with limited resources.
